# Soft Active Polymers for Biomimetic Shape Morphing Wings

**DOI:** 10.3390/biomimetics11030189

**Published:** 2026-03-05

**Authors:** Chao Yuan, Changyue Liu, Zhijian Wang

**Affiliations:** 1 School of Materials Science and Engineering, Beihang University, Beijing 100191, China; yuanchao2025@buaa.edu.cn (C.Y.); liuchangyue@buaa.edu.cn (C.L.); 2 AVIC Manufacturing Technology Institute, Beijing 100024, China

**Keywords:** shape morphing wings, soft active materials, shape memory polymers, dielectric elastomers, liquid crystal elastomers

## Abstract

In nature, avian species achieve remarkable aerodynamic efficiency by seamlessly coordinating flexible soft tissues to create continuous, adaptive wing surfaces, significantly minimizing drag and eliminating parasitic turbulence. Traditional shape morphing systems rely on bulky mechanical linkages that add excessive weight, often offsetting aerodynamic gains. The integration of soft active materials has emerged as a transformative solution for weight-efficient, seamless actuation. However, a significant disconnect remains between laboratory-scale research and practical aerospace implementation. This perspective evaluates three prominent classes of soft active materials, shape memory polymers (SMPs), dielectric elastomers (DEAs), and liquid crystal elastomers (LCEs), analyzing their actuation mechanisms and comparing their performance in load-bearing, response bandwidth, and energy efficiency. By addressing the necessity of structural-material synergy, we discuss the potential solution for bridging the gap between material synthesis and system-level flight performance to enable the successful deployment of soft active materials in future aerial platforms.

## 1. Introduction

Conventional aircraft are optimized for a specific design point, leading to massive inefficiencies during takeoff, landing, and maneuvering. With the rapid advancement of aerospace technology, aircraft are facing increasingly stringent performance requirements, for example, optimal aerodynamic performance across the entire flight envelope. While the birds can alter their geometry in response to changing flight environments, profiles, and mission objectives. This adaptability allows for highly maneuverable and flexible control over flight paths, altitudes, and speeds. Mimicking these biological mechanisms, shape-morphing wings represent a promising direction for advanced aviation [1]. Global morphing, such as variable sweep and wing folding, allows aircraft to dynamically alter their aerodynamic layout, improving adaptability across vast altitudes and speed regimes for multi-mission use. Conversely, local morphing, including camber variation and wingtip folding, offers civil aviation significant gains in lift-to-drag ratios, fuel economy, and noise suppression, directly supporting the push toward green aviation [2,3]. By dynamically modulating camber, twist, and span, these systems enable aircraft to minimize drag, reduce carbon emissions, and significantly expand their operational envelopes.

Based on the deformation characteristics of flexible skins in different wing deformation forms, they can be classified into curved skins, tensile/compressive skins, shear skins, three-dimensional deformable skins, etc. [4]. Among them, curved skins maintain a constant contour length and primarily undergo bending deformation during deformation; shear skins require shear deformation to accommodate changes in the wing surface geometry; while three-dimensional deformable skins are required to achieve complex deformations that combine stretching/compression along the span and chord directions, as well as in-plane shear. The concept of curved skin was first introduced by the Deutsches Zentrum für Luft-und Raumfahrt (DLR) in the Smart Leading Edge Device (Smart LED) project, indicating that variable camber leading edge skin can be achieved through bending deformation, rather than in-plane tensile/compressive deformation [5]. This fundamental discovery provided core criteria for subsequent designs of variable camber leading edge skin, and curved skin driven by multiple internal drive points has become the main structural form of variable camber leading edge skin since then. NextGen Company in the United States introduced the concept of shear skin in the Morphing Aircraft Structures (MAS) project, proposing a bat-shaped morphing wing with a large sweep angle variation from 15° to 60°, and a wing area change of over 50%. In the Active Flexible Trailing Edge Flaps (ACTE) project, the American company Flexsys proposed a three-dimensional deformable skin solution that utilizes a transition structure resembling a folding fan to achieve a seamless connection between the deformable and fixed sections of the wing [6]. The developed FlexFoil™ deformable wing, mounted on a Gulfstream III business jet, successfully completed flight tests at 0.75 MPa, achieving smooth and continuous trailing edge deformation from −9° to 40°, and achieving a noise reduction effect of 30% [7].

Historically, morphing has been achieved through discrete mechanics, heavy hydraulic actuators, complex mechanical linkages, and sliding surfaces. These bulky mechanisms significantly increase the weight of the aircraft, which offsets the aerodynamic gains. Additionally, complex design and control lead to increased failure points and rigorous maintenance schedules. In nature, avian species achieve sophisticated shape transformation through the seamless coordination of flexible soft tissues, such as musculature, tendons, and elastic dermis, rather than through discrete mechanical joints. This biological integration facilitates a continuous, adaptive wing surface that effectively minimizes aerodynamic drag and eliminates the parasitic turbulence.

To biomimetic this efficiency, soft active polymeric materials have emerged as a transformative class of soft active shape morphing materials [8,9]. These materials are capable of undergoing large deformations in response to specific external stimuli, such as thermal heating, electric fields, or light irradiation. By transducing external stimuli directly into mechanical work at the molecular level, these polymers function as both sensor and actuator. Over the past several decades, the field has seen a surge in the development of these materials. Integrating soft active polymers into aerospace design could catalyze a paradigm shift. Indeed, active morphing materials are now viewed as the promising structural solution for the next generation of truly adaptive aircraft.

However, a significant disconnect remains between current material research and practical aerospace applications. To date, most extant soft active materials have been designed for low-power, small-scale soft robotics, facilitating tasks such as crawling, rolling, or undulatory locomotion in controlled laboratory environments [10,11,12,13,14]. In contrast, the requirements for shape morphing in aerial vehicles are fundamentally more demanding, which necessitates a suite of properties that are often mutually exclusive in traditional material design. For example, an aircraft wing must maintain a precise aerodynamic profile against intense aerodynamic loading, which requires materials that possess both high elastic moduli and significant force output. This creates a critical stiffness paradox: the morphing structure must strike a delicate balance between being compliant enough to undergo large-scale, reversible deformation and being robust enough to carry the primary structural loads of the airframe.

Beyond structural integrity, the temporal and environmental demands of flight further widen the gap between lab-scale polymers and flight-ready hardware. While a rolling robot can operate with slow actuation cycles, an aircraft requires rapid, high-frequency adjustments to effectively mitigate atmospheric gusts and maintain stability during high-speed maneuvers. This necessitates a high-bandwidth response that many current stimuli-responsive polymers have yet to achieve at scale. Furthermore, aerial materials must function reliably under an extreme temperature range, while resisting degradation from UV exposure and moisture. Because these harsh conditions are seldom addressed in typical soft robotics research, it is emerging to clarify the shape change mechanisms and performance of the soft active polymeric materials and requirements for the aerial wings. In the following, we will introduce recent advancements of soft active materials in the shape morphing aerial wings and discuss the pros and cons of different soft active polymers. It is worth noting that the metrics such as dynamic pressure, wing loading, actuation stress, and the required strain rates for alleviation are critical. However, these values vary by orders of magnitude depending on the specific aerial vehicle and mission profile. Consequently, concluding these disparate requirements into a single “optimal design” graph remains a significant challenge. The scope of this manuscript focuses specifically on soft active polymeric materials for shape morphing wings.

## 2. Brief Introduction of Soft Active Materials for Aerial Wings

Selecting the appropriate material for an aerial morphing wing necessitates a careful balance between actuation performance and structural integrity. Recent research has pivoted toward the integration of shape memory polymers (SMPs), dielectric elastomers (DEAs), and liquid crystal elastomers (LCEs) as potential candidates for adaptive wing architectures [15]. Each of these material classes presents a unique profile of trade-offs regarding stiffness, response latency, and energy efficiency. In the following sections, we evaluate their respective morphing mechanisms and the critical properties that define their suitability for flight applications.

### 2.1. Shape Memory Polymers (SMPs)

**Figure 1:**
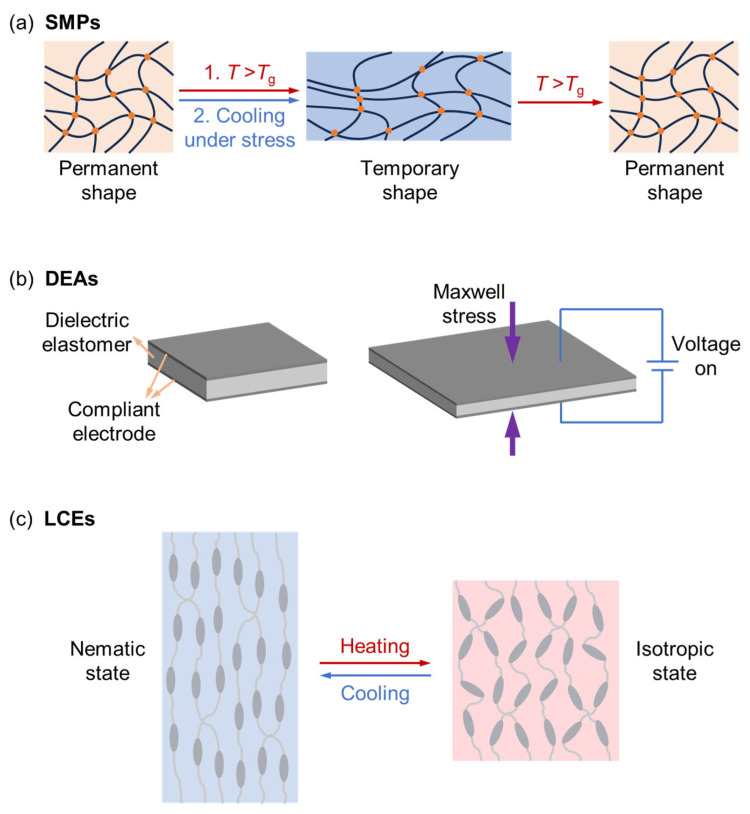
The actuation mechanisms of (**a**) SMPs, (**b**) DEAs, and (**c**) LCEs.

SMPs are a class of stimuli-responsive materials that can maintain a stable ‘temporary’ shape and recover a pre-determined ‘permanent’ shape upon exposure to an external trigger, most commonly heating (Figure 1a) [16,17,18,19]. The transition is governed by switching the chemical or physical cross-links, including the glass transition, crystalline phase, hydrogen-bonding, metal-ligand interaction, etc. In the context of aerospace morphing, the primary advantage of SMPs lies in their dramatic modulus change. Take the glassy polymers as an example. Below the glass transition temperature, the polymer is in a glassy state, providing the high elastic modulus (often in the GPa range) required to carry primary structural loads and resist aerodynamic pressures. When heated above the glassy transition temperature, the material transits to a rubbery state, becoming highly compliant and allowing for significant deformation with minimal actuation force. This stiffness-on-demand makes SMPs an excellent candidate for load-bearing morphing components. However, their application is often tempered by thermal diffusion, leading to relatively slow response times and high power consumption for maintaining the active state. Additionally, when heated, the modulus of SMPs will dramatically drop to the hundreds or tens of MPa range, leading to aerodynamic concerns. Recent research has therefore focused on SMP composites (SMPCs), which integrate conductive fillers or fibers to improve both the mechanical strength and the speed of electro-thermal actuation.

The Cornerstone Research Group (CRG) Company developed a fabrication technique for seamless skins based on SMPCs [20]. The wing skin utilizes SMPCs at the joints to enable deformation during the folding or unfolding of the hinges. At elevated temperatures, the SMPC material transitions into a rubbery elastic state, allowing the seamless skin to be folded without structural damage. Upon reheating, the material exhibits its shape memory effect, returning to its original configuration. Yu et al. [21] proposed a concept for morphing wings composed of SMPs and SMCs. They compared the deployment processes of carbon fiber-reinforced SMPC skins with those reinforced by shape memory alloy (SMA) wires and elastic steel sheets. The results indicated that SMPC skins reinforced by SMA wires and elastic steel sheets exhibit higher recovery speeds than those reinforced solely by carbon fiber. The shape morphing wings with SMPCs showed excellent deformation properties. However, during thermal actuation, the stiffness of SMPs decreases significantly as the temperature exceeds the glass transition point, raising concerns regarding their ability to withstand aerodynamic loads. To address this, common strategies involve incorporating high-strength fibers or integrating reinforcing structures to enhance composite stiffness. Both have demonstrated substantial improvement in structural integrity.

While SMPs exhibit impressive deformation capabilities, a significant limitation lies in their inherent one-way shape memory effect. In a standard thermomechanical cycle, the material can be programmed into a temporary shape and will return to its original ‘permanent’ configuration upon heating. However, once the stimulus is removed, the polymer remains in that recovered state. It cannot autonomously return to the temporary shape without the re-application of an external mechanical force. This irreversibility poses a substantial hurdle for aerospace applications, where cyclic morphing is required. In a wing structure, a one-way SMP would necessitate a complex setup to reset the wing to its morphed position after each cycle. This requirement often introduces the very mechanical complexity that soft active materials were intended to eliminate.

To address this, two-way SMPs (2W-SMPs) have emerged as a superior alternative, capable of autonomous and reversible shape alteration simply by cycling the temperature [22,23,24]. The earliest iterations of 2W-SMPs leverage the properties of semi-crystalline polymer networks characterized by two distinct crystalline phases with disparate melting temperatures (*T*_m,1_ and *T*_m,2_). In this dual-phase system, the high-melting-point crystalline phase (*T*_m,2_) acts as a permanent physical cross-link, maintaining the network’s structural integrity and remembering the shape. When the material is heated to a temperature between the two melting points (*T*_m,1_ < *T* < *T*_m,2_), the low-melting-point phase transitions into an amorphous state, allowing for deformation. Upon subsequent cooling, the recrystallization of the low-temperature phase is guided by the internal stress fields generated by the intact high-temperature skeleton. This internal stress-induced crystallization forces the polymer to return to its temporary shape autonomously, effectively providing a bistable and self-programming solution for reversible wing reconfiguration without the need for external mechanical intervention. However, when heated above the *T*_m,1_, both crystalline phases will be melted, erasing the reversible actuation performance. Thus, the cycling temperature range must be carefully designed.

### 2.2. Dielectric Elastomers (DEAs)

While SMPs provide the structural rigidity necessary for heavy load-bearing applications, DEAs have garnered significant interest for their capacity for rapid, high-strain actuation that closely mimics the functionality of biological muscles. A typical DEA comprises a thin, highly compliant elastomer film sandwiched between two compliant electrodes. When an external electric field is applied, the resulting electrostatic attraction, known as Maxwell stress, compresses the film in thickness and expands it in area, facilitating large-scale deformation (Figure 1b) [25,26,27,28]. These actuators offer distinct advantages with their high-frequency response, significant actuation strain, and high energy efficiency. Unlike thermally driven polymers that suffer from inherent thermal lag, DEAs can operate at frequencies exceeding 100 Hz, rendering them uniquely suited for active aeroelastic control tasks such as flutter suppression or millisecond-range gust alleviation.

Furthermore, upon the application of an electric voltage, DEAs can achieve area strains well over 100%, enabling radical geometry changes [29,30,31]. Beyond actuation, these materials can maintain a morphed state with minimal power consumption and provide integrated self-sensing capabilities by monitoring changes in capacitance during the deformation process. The exceptional energy density and power-to-weight ratio of DEAs have also made them a promising candidate in the development of micro-aerial flapping-wing robots.

Recent advancements have further demonstrated their ability to survive millions of cycles, enabling the endurance of autonomous flapping-wing platforms. By integrating these actuators directly into the wing spar, all-soft aerial systems are developed that are not only agile but inherently resilient to collisions. It offers a practical pathway for robust, bio-inspired flight in complex environments. For example, researchers have demonstrated flying insect inspired wings that can maintain aerodynamic lift while providing the rapid stroke necessary for agile maneuverability [29]. Nevertheless, the softness of DEAs arises as a big concern for the aerial shape morphing wings with a large size. By integrating stiff carbon-fiber frames into DEAs or using stacked actuator configurations, the drawbacks in softness of pure elastomers can be tackled. However, the requirement for high-voltage power electronics (often in the kilovolt range) remains a primary challenge for compact aerial platforms.

### 2.3. Liquid Crystal Elastomers (LCEs)

Liquid crystal elastomers (LCEs) represent a class of morphing polymers synthesized by integrating rigid liquid crystalline mesogens into a cross-linked polymer network [32,33,34]. These materials are capable of undergoing a reversible phase transition from a highly ordered liquid crystalline phase to a disordered isotropic phase upon heating, a transition that triggers significant macroscopic shape change as the mesogens lose their alignment (Figure 1c) [35,36,37]. A primary advantage of LCEs in shape-morphing applications is their intrinsic two-way shape memory effect, which distinguishes them from traditional SMPs. Unlike SMPs, which require a secondary mechanical programming step to set a temporary shape, LCEs autonomously return to their original configuration upon cooling. It is because, upon cooling, the elastic energy stored in the polymer network during the initial shape change acts as an internal bias, driving the mesogens back to their initial state. Furthermore, the specific mode of deformation, whether contraction, bending, or complex wing twisting, is directly dictated by the initial alignment pattern of the mesogens, allowing for unparalleled control over complex aerodynamic geometries. By incorporating photothermal or electrothermal additives, these materials can be triggered remotely by light or electric signals to produce precise, anisotropic contractions tailored to specific flight conditions.

Despite their programmable elegance and autonomous reversibility, there is no reported prototype shape morphing wings made from LCE materials. Several critical hurdles remain for the deployment of LCEs in high-load aerospace environments. The preparation of aligned LCE structures currently necessitates a complex two-step process to fix the mesogen orientation [38,39]. The available chemical space for these materials remains largely restricted to thiol-acrylate Michael addition reactions. This synthetic limitation often results in materials that suffer from low force output and limited mechanical robustness, which are currently insufficient for resisting the intense aerodynamic pressures encountered during flight. To bridge this gap, there is an emergent need to develop robust LCE architectures that combine simplified manufacturing processes with enhanced mechanical properties. Future research must prioritize the development of high-strength LCE composites that can provide the necessary structural integrity while maintaining the precise, multi-modal deformation that makes this class of materials so promising for the next generation of adaptive aircraft.

## 3. Comparison of Soft Active Material Candidates

Selecting the most suitable polymeric candidate for an adaptive wing requires a rigorous assessment of the trade-offs between mechanical strength, actuation speed, and operational reversibility. SMPs stand out for their superior load-bearing capacity and high stiffness in the temporary state, which is essential for resisting aerodynamic pressures. However, they are fundamentally hindered by slow thermal response times and a predominantly one-way actuation cycle that necessitates an external mechanical force for resetting. In contrast, DEAs function as high-bandwidth artificial muscles capable of millisecond-range adjustments and massive area strains, making them ideal for active gust alleviation and high-frequency flutter control. Nevertheless, their inherent softness makes them prone to aeroelastic instability under load, and their reliance on bulky high-voltage power electronics complicates the overall weight budget of the airframe. LCEs offer a compelling alternative through their intrinsic two-way reversibility and programmable anisotropy, which allows for complex deformations like wing twisting without the secondary programming steps required by SMPs.

Another critical property which are neglected is energy efficiency, as it dictates the power requirements and thermal management needs of the overall aircraft system. In this regard, DEAs are widely considered the most efficient, often achieving electromechanical conversion efficiencies between 60% and 90%. Because they function as variable capacitors, DEAs store electrical energy during actuation, a portion of which can theoretically be recovered during the relaxation phase. Furthermore, they require negligible power to maintain a static displacement, drawing current only to compensate for minor leakage.

In contrast, SMPs and LCEs exhibit significantly lower operational efficiency, typically well below 1%. This is primarily because they rely on thermal stimuli to trigger phase transitions. A vast majority of the input energy is dissipated as waste heat to the surrounding environment rather than being converted into mechanical work. Additionally, a distinction must be made between actuation efficiency and holding efficiency in the shape morphing wings. While SMPs are inefficient during the transition phase, they are exceptionally efficient at holding a shape, as they require zero power once the material cools and locks into its glassy state. DEAs and LCEs, by comparison, may require continuous power to sustain a morphed geometry if the structure lacks mechanical bi-stability. Therefore, the choice of material involves a trade-off. DEAs are superior for high-frequency, energy-efficient movement, whereas SMPs are the most energy-efficient choice for long-duration deployments where the wing must remain in a fixed, morphed configuration for extended flight segments.

We summarize the comparison metric of the performance of the soft active materials for aerospace morphing in the following Table 1.

## 4. Perspective

While mechanism-based shape morphing has successfully demonstrated substantial improvements in aerodynamic efficiency, these bulky systems significantly increase the aircraft’s structural weight, often offsetting the gains achieved through morphing. Furthermore, the reliance on intricate mechanical linkages and actuators necessitates complex control architectures, leading to a higher density of potential failure points and more rigorous maintenance requirements. Soft active polymer materials offer a compelling alternative. They enable truly continuous shape morphing and eliminate the mechanical instabilities associated with discrete components, providing a pathway toward lighter and more reliable adaptive wings. However, the materials for shape-morphing wings must withstand significant aerodynamic structural loads, necessitating the simultaneous consideration of structural integrity, actuation authority, and fluid dynamics. In addition, the optimal design methodologies highly depend on the specific aerial vehicle and mission profile. It remains significant challenging to establish a universal design framework. It is a primary frontier to bridge the gap between materials capabilities and flight-ready performance.

As we discussed above, three typical soft active polymeric materials, such as SMPs, DEAs, and LCEs, each offer distinct advantages. However, no single material currently satisfies the full spectrum of aerospace requirements, which range from high structural rigidity to millisecond response times. It is essential to recognize that a morphing wing is not merely an assembly of smart materials, but a sophisticated integration of geometry, structural mechanics, actuation methods, and material response. Transitioning these polymers from laboratory prototypes to flight-ready aerial wings necessitates bridging the critical divide between molecular material science and structural aerospace engineering. Therefore, the strategic design of the wing architecture is of paramount importance, where the choice of actuation, whether electrical, thermal, or fluidic driven, is dictated by the specific shape-morphing requirements and operational constraints of the mission. The core challenge lies in synergizing material-level stimuli-responsiveness with system-level structural integrity to achieve a cohesive, high-performance aerodynamic effect.

The most immediate path toward resolving the stiffness paradox is the engineering of hybrid active composites, creating a biomimetic skeleton-and-muscle architecture. By combining the high-load-carrying capacity and state-locking functions of SMPs or LCEs with the rapid, muscle-like response of DEAs, researchers can effectively decouple structural integrity from actuation speed. In this framework, the SMP or LCE component acts as a variable-stiffness skeleton that maintains the optimized aerodynamic profile, while the DEA provides the high-bandwidth reflexes necessary for real-time gust suppression. Such a system allows for a truly adaptive airframe capable of surviving high dynamic pressures while maintaining the agility of a biological flyer.

Furthermore, the advancement of manufacturing techniques will be crucial for scaling these materials from lab-scale specimens to full-sized wing components. Advanced additive manufacturing allows for the spatial programming of material properties, such as varying the cross-link density or the alignment of liquid crystal mesogens, within a single, continuous structure. This enables the fabrication of functionally graded wings that are compliant at the leading edge for seamless morphing, yet transition into high-stiffness regions at the wing for primary load bearing.

As these materials become more complex and responsive, the focus must shift toward closed-loop, AI-driven control systems. Because soft active materials often exhibit non-linear behavior and hysteresis, traditional control laws are frequently insufficient for precise maneuvers. Future aircraft will require integrated sensors, leveraging the self-sensing capabilities, coupled with machine learning algorithms that can predict and compensate for aerodynamic changes in real time.

Finally, the industry must standardize the fatigue life and environmental resilience of these polymers to meet stringent aviation safety certifications. The goal is no longer just to build a wing that moves, but a wing that feels and adapts as a living bird. By bridging the gap between molecular-level material science and system-level flight control, we anticipate a vision of biomimetic aircraft that do not merely fly through the air, but actively interact with and adapt to the atmosphere with the elegance and efficiency of a biological entity.

The development of shape-morphing wings utilizing soft active polymeric materials holds immense potential for the future of aviation. However, substantial hurdles remain. For example, critical metrics, such as dynamic pressure, wing loading, actuation stress, and the strain rates required for gust alleviation, vary by several orders of magnitude depending on the specific aerial vehicle and its mission profile. Consequently, satisfying these disparate requirements into a single, unified “optimal design” framework remains a significant challenge. Overcoming these barriers necessitates a deep interdisciplinary corporation, particularly in structural design, to effectively integrate multi-functional materials into a cohesive system. We anticipate that as these material-structure synergies mature, they will enable a new generation of aircraft capable of unprecedented levels of efficiency and adaptability.

## Figures and Tables

**Table 1:** Comparison of soft active polymeric materials for aerospace morphing.

Material Class	Actuation Speed	Modulus	Reversibility	Conversion Efficiency	Energy Source	Holding Power
SMPs	Low (s to min)	Very high	Usually one-way	Very low (<1%)	Thermal	Zero (state-locked)
DEAs	Very high (ms)	Low	Yes (instantaneous)	High (60~90%)	Electrical	Very low (leakage only)
LCEs	Moderate	Moderate	Yes (intrinsic two-way)	Very low (<1%)	Thermal/light	High (requires constant stimulus)

## Data Availability

No new data were created or analyzed in this study.
